# Mechanical Properties of Cu_2_O Thin Films by Nanoindentation

**DOI:** 10.3390/ma6104505

**Published:** 2013-10-11

**Authors:** Sheng-Rui Jian, Guo-Ju Chen, Wei-Min Hsu

**Affiliations:** Department of Materials Science and Engineering, I-Shou University, Main Campus: No.1, Sec. 1, Syuecheng Rd., Dashu District, Kaohsiung City 84001, Taiwan; E-Mails: gjchen@isu.edu.tw (G.-J.C.); cdjhs50113@yahoo.com.tw (W.-M.H.)

**Keywords:** Cu_2_O thin film, XRD, AFM, SEM, nanoindentation, hardness

## Abstract

In this study, the structural and nanomechanical properties of Cu_2_O thin films are investigated by X-ray diffraction (XRD), atomic force microscopy (AFM), scanning electron microscopy (SEM) and nanoindentation techniques. The Cu_2_O thin films are deposited on the glass substrates with the various growth temperatures of 150, 250 and 350 °C by using radio frequency magnetron sputtering. The XRD results show that Cu_2_O thin films are predominant (111)-oriented, indicating a well ordered microstructure. In addition, the hardness and Young’s modulus of Cu_2_O thin films are measured by using a Berkovich nanoindenter operated with the continuous contact stiffness measurements (CSM) option. Results indicated that the hardness and Young’s modulus of Cu_2_O thin films decreased as the growth temperature increased from 150 to 350 °C. Furthermore, the relationship between the hardness and films grain size appears to closely follow the Hall-Petch equation.

## 1. Introduction

Cuprous oxide (Cu_2_O), a P-type semiconductor with a direct bandgap of ~2 eV [1], has attracted much interest because of its potential applications for thin film hetero-junction solar cells [2,3] and sensors [4]. However, most of the studies reported to date have been focused on the optical and electrical properties of Cu_2_O thin films [1,2,3,4], and little attention has been devoted to the mechanical properties of Cu_2_O films. Since the rapid development of the field of nanotechnology over the last decade, there has been an increasing demand for handling materials at nanoscale for making nanostructures and/or nano-devices. Therefore, it has become crucially important to understand how the materials behave at the nanometer-scale. In particular, it has been well established that, when the size is down to the nanoscale, the mechanical properties of materials, for example the hardness and Young’s modulus, may be very different from that in the bulk form. In this respect, a precise measurement of the mechanical properties of materials is required to use them as structural elements in nano-devices.

Nanoindentation is a unique and powerful technique for investigating the mechanical characteristics of thin films [5,6,7] and various materials within the submicron-or nano-scales [8,9,10]. This study is therefore focused on nanomechanical characterizations of Cu_2_O thin films deposited on the glass substrates using radio frequency sputtering system at various growth temperatures by means of nanoindentation technique. The structure and surface morphology of Cu_2_O thin films are characterized by using X-ray diffraction (XRD), atomic force microscopy (AFM) and scanning electron microscopy (SEM) techniques. Hardness and Young’s modulus of Cu_2_O thin films were measured by means of Berkovich nanoindentation operating with the continuous stiffness measurement (CSM) mode [11]. Changes in mechanical properties for Cu_2_O thin films are discussed in conjunction with the growth temperature and the average grain size.

## 2. Experimental Details

In this work, Cu_2_O thin films were prepared by radio frequency magnetron sputtering. The films were grown on Corning 1737 glass substrates at temperature range of 150 to 350 °C for 30 min. Sputtering power of 50W was delivered to the Cu target. The base pressure was 5 × 10^−6^ torr. Gas mixture of Ar and O_2_ with flow ratio of 20:1 was used as the working atmosphere and the working pressure fixed at 4 mtorr during growth. All of Cu_2_O thin films are about 1 μm thick.

The crystal structure of Cu_2_O thin films were analyzed by means of X-ray diffraction [Panalytical X’Pert XRD (PANalytical, Almelo, The Netherlands) CuKα, λ = 1.5406 Å]. A scanning electron microscopy (SEM, Hitachi S-4700, Tokyo, Japan) is used to analyze the cross-sectional structure of Cu_2_O thin films. In addition, the surface features were examined by atomic force microscopy (AFM; Topometrix-Accures-II, Topometrix Corporation, Santa Clara, CA, USA). The root mean square of the surface roughness, *R*_rms_, was calculated by the following Equation [12]:

(1) $$R_{\text{rms}}={\left(\frac{1}{N}\sum_{n=1}^{N}r_{n}^{2}\right)}^{1/2}$$

Here, *N* is the number of data and *r_n_* is the surface height of the *n*th datum.

Nanoindentation experiments were preformed on a MTS Nano Indenter^®^ XP system (MTS Cooperation, Nano Instruments Innovation Center, Oak Ridge, TN, USA) with a three-sided pyramidal Berkovich indenter tip by using the continuous stiffness measurement (CSM) technique [11]. This technique is accomplished by imposing a small, sinusoidal varying force on top of the applied linear force that drives the motion of the indenter. The displacement response of the indenter at the excitation frequency and the phase angle between the force and displacement are measured continuously as a function of the penetration depth. Solving for the in-phase and out-of-phase portions of the displacement response gives rise to the determination of the contact stiffness as a continuous function of depth. As such, the mechanical properties changing with respect to the indentation depth can be obtained. The nanoindentation measurements were carried out as follows. First, prior to applying loading on Cu_2_O thin films, nanoindentation was conducted on the standard fused silica sample to obtain the reasonable range (the Young’s modulus of fused silica is 68–72 GPa). Then, a constant strain rate of 0.05 s^−1^ was maintained during the increment of load until the indenter reached a depth of 60 nm into the surface. The load was then held at the maximum value of loading for 10 s in order to avoid the creep which might significantly affect the unloading behavior. The indenter was then withdrawn from the surface at the same rate until the loading has reduced to 10% of the maximum load. Then, the indenter was completely removed from the material. In this study, constant strain rate was chosen in order to avoid the strain-hardening effects. At least 30 indentations were performed on each sample and the distance between the adjacent indents was kept at least 5 μm apart to avoid interaction.

In indentation measurement, the hardness is defined as the applied indentation load divided by the projected contact area, *H* = *P*_max_/*A_c_*, where *A_c_* is the projected contact area between the indenter and the sample surface at the maximum indentation load, *P*_max_*.* For a perfectly sharp Berkovich indenter, the projected area is given by $A_{c}=24.56h_{c}^{2}$ with *h_c_* being the contact depth.

The elastic modulus of the sample can be calculated based on the relationships developed by Sneddon [13]: $S=2\beta E_{r}\sqrt{A_{p}}/\sqrt{\pi }$. Here, *S* is the contact stiffness of the material and β is a geometric constant with β = 1.00 for Berkovich indenter, respectively. The reduced elastic modulus, *E_r_*, can be calculated from the following Equation:

(2) $$E_{r}={\left(\frac{1-v_{\text{film}}^{2}}{E_{\text{film}}}+\frac{1-v_{i}^{2}}{E_{i}}\right)}^{-1}$$

Here, *v* is the Poisson’s ratio and the subscripts *i* and *film* denote the parameters for the indenter and measured thin films, respectively. For diamond indenter tip, *E_i_* = 1141 GPa, *v_i_* = 0.07 and, *v*_film_ = 0.3 is assumed for Cu_2_O thin films in this work.

## 3. Results and Discussion

**Figure 1:**
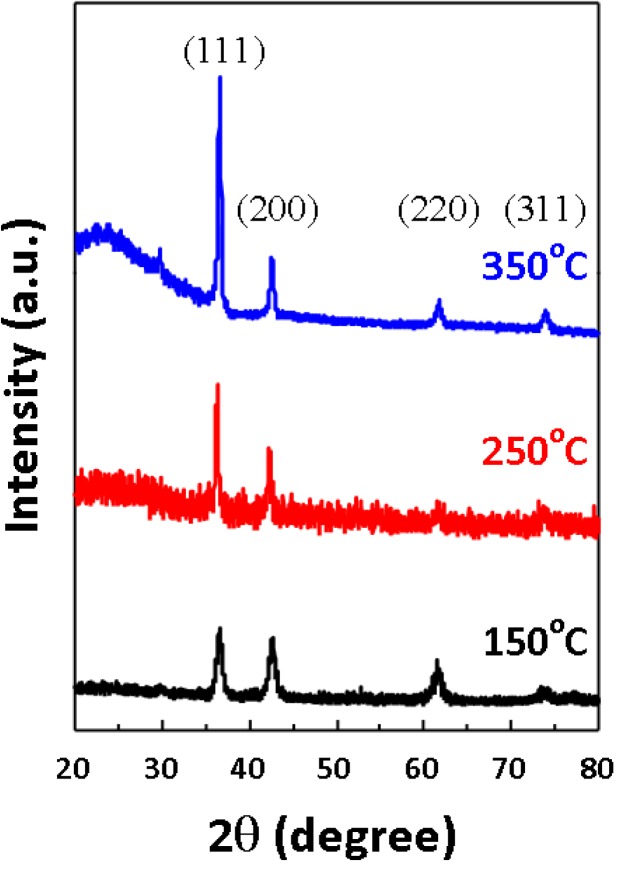
X-ray diffraction (XRD) patterns of Cu_2_O thin films deposited at various growth temperatures of 150 °C, 250 °C and 350 °C, respectively.

The XRD results of Cu_2_O thin films obtained with various growth temperatures of 150, 250 and 350 °C are shown in Figure 1. It is evident that the intensity and the full width at half maximum (FWHM) of the Cu_2_O (111) diffraction peak are both improved with the increasing growth temperature, indicating a tendency of better film crystallinity and increased films grain size. The grain size (*D*) can be estimated according to Scherrer’s equation [14], *D* = 0.9λ/(*B*cosθ). Here, λ, *B* and θ denote the X-ray wavelength, the FWHM of (111) peak, and the corresponding Bragg’s diffraction angle, respectively. The estimated mean grain sizes of Cu_2_O thin films are 34.5 ± 0.8, 78.6 ± 0.5 and 102.4 ± 0.2 nm for the various growth temperatures of 150, 250 and 350 °C, respectively.

**Figure 2:**
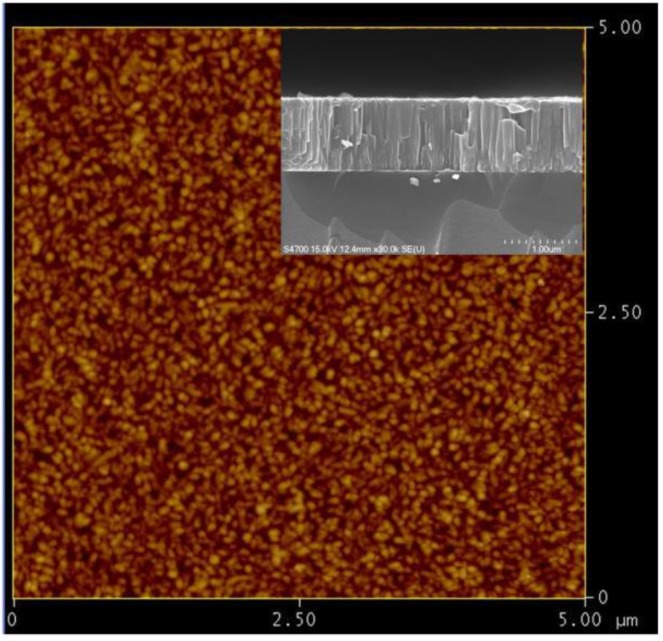
Atomic force microscopy (AFM) image of Cu_2_O thin film deposited at the growth temperature of 350 °C. The inset figure shows the corresponding cross-sectional scanning electron microscopy (SEM) image.

Figure 2 displays a typical AFM image of Cu_2_O thin film deposited at 350 °C. In Figure 2, it can be found that the radio frequency magnetron sputtering derived Cu_2_O thin film exhibit dense microstructures with the homogenous grain size. As the growth temperature increases from 150 to 350 °C, the average surface roughness of Cu_2_O thin films increases from 2.8 ± 0.6 to 8.7 ± 0.1 nm. The higher growth temperatures lend more thermal energy to activate atom diffusion and, hence, facilitate the repairing the dislocated atomic occupancies and even promote the coalescence of adjacent grains. As can be seen in the inset of Figure 2, the films with the columnar structures are significantly displayed in the cross-sectional SEM image. Consequently, the major grain growth is expected to result in marked increase in both the grain size and the surface roughness of the resultant films. The average surface roughness and the grain size of Cu_2_O thin films are summarized in Table 1.

**Figure 3:**
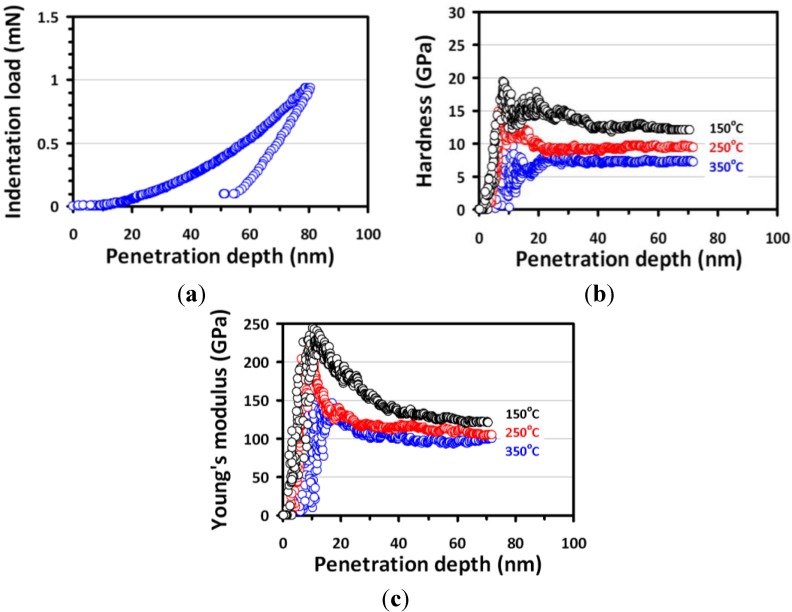
(**a**) A typical load-displacement curve for Cu_2_O thin films deposited at 350 °C. (**b**) The hardness-displacement curves. (**c**) Young’s modulus-displacement curves for Cu_2_O thin films deposited at various growth temperatures.

The load-displacement curve for Cu_2_O thin film deposited at 350 °C, which reflects the general deformation behavior during the penetration of a Berkovich indenter loaded with CSM mode, is shown in Figure 3a. The load-displacement response obtained by nanoindentation contains information about the elastic behavior and plastic deformation, thus can be regarded as the “fingerprint” of the properties of Cu_2_O thin films. The curve appears to be smooth and regular. The absence of any discontinuities along either the loading or unloading segment is in sharp contrast to those observed in GaN thin films [5,7,15] and in single crystal Si [16], indicating that no pressure-induced phase transformation is involved here.

Figure 3b,c present the hardness and Young’s modulus* versus* penetration depth curves for Cu_2_O thin films deposited at 150, 250 and 350 °C, respectively. The hardness as a function of penetration depth behaves similarly in all cases, indicating that similar indentation-induced deformation mechanism is prevailing in all films studied. The initial sharp increase in hardness at small penetration depth is usually attributed to the transition between purely elastic to elastic/plastic contact [17]. Only under the condition of a fully developed plastic zone does the mean contact pressure represent the hardness [18]. When there is no plastic zone, or only a partially formed plastic zone, the mean contact pressure measured according to the Oliver and Pharr method [19] is usually smaller than the nominal hardness. After the first stage, the hardness decreases with increasing depth in a rather meandering manner, presumably involving massive dislocation and grain boundary activities relevant to the fine grain structure of the films. Nevertheless, the fact that it eventually reaches a constant value at a moderate indentation depth indicates that a single material is being measured. The hardness values obtained at this stage, thus, can be regarded as the intrinsic properties of the films. As can be seen in Figure 3c, the penetration depth dependence of the Young’s modulus behaves similarly as that of hardness. Consequently, both mechanical parameters were determined using the curves obtained from the CSM loading scheme (Figure 3b,c) by taking the average values within the penetration depth of 40–70 nm. It is generally accepted that the indentation depth should never exceed 30% of the film thickness to avoid the substrate effect on hardness and modulus measurements [8]. Consequently, the chosen indentation depth was deep enough to observe the plastic deformation during nanoindentation yet to be shallow enough to reduce the effects of surface roughness [20] and substrate [8]. Table 1 summarizes the hardness and Young’s modulus for Cu_2_O thin films obtained by nanoindentation. It is evident that the values of hardness and elastic modulus of the present Cu_2_O thin films are substantially larger than those of Cu_2_O nanocubes, namely, 0.62 ± 0.2 and 82 ± 12 GPa for hardness and elastic modulus, respectively [21]. In particular, the hardness of films is order of magnitude higher than that of Cu_2_O bulks, indicating the prominent role played by microstructures here.

**Table 1:** Grain size, surface roughness, hardness and Young’s modulus of Cu_2_O thin films.

Growth temperature	*D* (nm)	*R*_rms_ (nm)	*H* (GPa)	*E*_film_ (GPa)
150 °C	34.5 ± 0.8	2.8 ± 0.6	12.3 ± 0.5	126.8 ± 4.8
250 °C	78.6 ± 0.5	4.5 ± 0.4	9.4 ± 0.3	112.4 ± 5.2
350 °C	102.4 ± 0.2	8.7 ± 0.1	7.2 ± 0.2	98.5 ± 6.9

**Figure 4:**
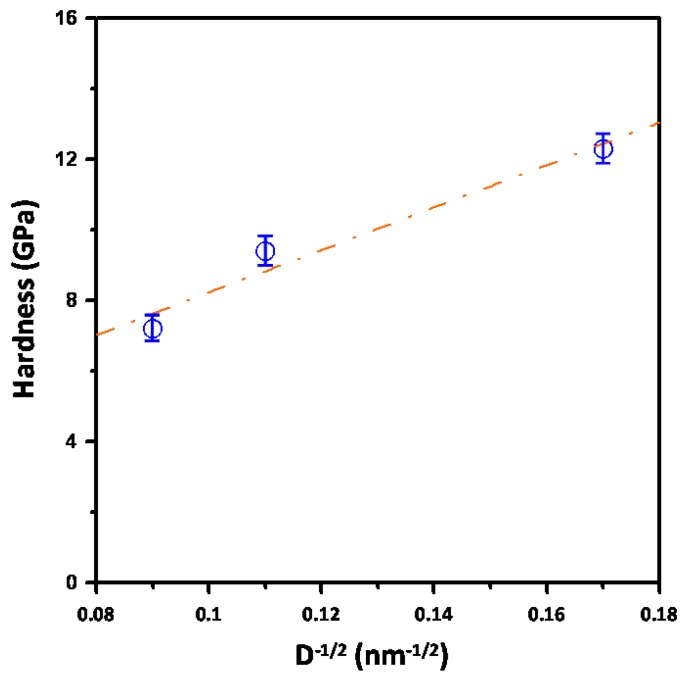
Plot of the experimental data of hardness* versus* the grain size. The dashed line is a fit to the data using the Hall-Petch equation with the form of *H*(*D*) = 1.39 + 64.46 *D*^−1/2^.

It is well known that the dependence of material hardness on the grain size can be described by the phenomenological “Hall-Petch” equation [22], as follows:

(3) $$H(D)=H_{0}+k_{\text{HP}}\;D^{-1/2}$$

where *H*_0_ and *k*_HP_ are denoted as the lattice friction stress and the Hall-Petch constant, respectively. In Figure 4, a plot of the hardness* versus D*^−1/2^ data for Cu_2_O thin films deposited at various temperatures is shown. We note that, although the grain size of Cu_2_O thin films remains relatively small compared to that of the usual metallic materials, the data still follow pretty closely to the Hall-Petch relation. In addition, the dashed line in Figure 4 is a fit to the experimental data using the Hall-Petch equation with the form of *H*(*D*) = 1.39 +64.46*D*^−1/2^. The obtained fitting parameter *H*_0_ = 1.39 GPa indicates a lattice friction stress relatively larger than the value obtained from Cu_2_O nanocubes, which might be due to more abundant lattice disorders in these films. On the other hand, the Hall-Petch constant of 64.46 GPa nm^1/2^ strongly indicates the effectiveness of grain boundary in hindering of dislocation movements in Cu_2_O thin flims.

Moreover, it is evident that both the hardness and Young’s modulus of Cu_2_O thin films decrease monotonically with increasing the growth temperature. The corresponding hardness (Young’s modulus) are 12.3 ± 0.5 (126.8 ± 4.9) GPa, 9.4 ± 0.3 (112.4 ± 5.2) GPa and 7.2 ± 0.2 (98.5 ± 6.9) GPa for Cu_2_O thin films deposited at 150, 250 and 350 °C, respectively. Since the higher growth temperature leads to larger grain size and better crystallinity for Cu_2_O thin films, as we have discussed previously, it is reasonable to consider that the decrease of hardness and Young’s modulus might be mainly due to the effects of improved crystalline quality and enhanced grain size induced by raising the film growth temperature [23].

## 4. Conclusions

In summary, a combination of XRD, SEM, AFM and nanoindentation techniques has been carried out to investigate the microstructural and nanomechanical properties of Cu_2_O thin films deposited at the various growth temperatures by using radio frequency magnetron sputtering. XRD and AFM consistently indicated that the grain size and crystalline quality of the Cu_2_O films were significantly improved with increasing deposition temperature. Nanoindentation results indicated that, depending on the grain size which is related to the growth temperature, Cu_2_O thin films have the hardness ranging from 7.2 ± 0.2 to 12.3 ± 0.5 GPa and Young’s modulus ranging from 98.5 ± 6.9 to 126.8 ± 4.9 GPa with the higher values being corresponding to lower growth temperature. The decrease of hardness with increasing growth temperature is mainly due to the effect of grain size. Indeed, by fitting the experimental hardness data to the Hall-Petch equation, a relatively large Hall-Petch constant of 64.46 GPa·nm^1/2^ is obtained, which strongly indicates the effectiveness of grain boundary in hindering of dislocation movements in Cu_2_O thin films. On the other hand, the slight decrease of Young’s modulus for films grown at higher temperatures is believed to be more relevant to the better crystalline quality of the films.
